# Sources, Status, and Potential Risks of Microplastics in Marine Organisms of the Bohai Sea: A Systematic Review

**DOI:** 10.3390/toxics13050400

**Published:** 2025-05-16

**Authors:** Jian Yang, Hongxia Li, Wei Ling, Yifei Li, Kangkang Zhang, Pu Zhang

**Affiliations:** 1 College of Civil and Transportation Engineering, Shenzhen University, Shenzhen 518060, China; y_jian0812@163.com; 2 National Engineering Research Center for Environment-Friendly Metallurgy in Producing Premium Non-Ferrous Metals, GRINM Resources and Environmental Technology Co., Ltd., Beijing 101407, China; 3 Beijing Key Laboratory of Resource-Oriented Treatment of Industrial Pollutants, University of Science and Technology Beijing, Beijing 100083, China; 4 Sinochem Environment Holdings Co., Ltd., Beijing 100071, China; lyf_33053085@163.com; 5 China Construction Industrial & Energy Engineering Group Co., Ltd., Nanjing 210046, China; zhangkangkang4134@cscec.com; 6 Central Research Institute of Building and Construction Co., Ltd., Beijing 100088, China; nuli123fendou456@sina.cn

**Keywords:** microplastics, Bohai Sea, aquatic organisms, traceability, risk management

## Abstract

This study focused on microplastic pollution in the Bohai Sea, employing bibliometric analysis and meta-integration methods to systematically analyze its pollution characteristics and ecological risks. The results indicated that microplastics primarily originated from land-based inputs (62%) and marine activities (23%). Microplastic concentrations in the Bohai Sea’s coastal areas were significantly higher than in deep waters, and the abundance of microplastics in aquaculture sediments was three to five times that in non-aquaculture areas. Bioaccumulation demonstrated a significant trophic magnification effect, with top predators containing much higher microplastic concentrations than plankton. The combined toxicity of microplastics and pollutants severely impacted key species, leading to a 92% decrease in Chinese shrimp populations and a significant reduction in benthic biodiversity. To address this issue, a “four-in-one” prevention and control system was proposed, encompassing source reduction, intelligent monitoring, targeted treatment, and regional collaboration, with measures including policy, technological innovation, and ecological restoration. This aims to provide scientific evidence for Bohai Sea ecological security management and offer a reference for microplastic management in globally semi-enclosed seas.

## 1. Introduction

Microplastics were typically defined as plastic particles with a diameter smaller than 5 mm, including fragments, fibers, particles, and microbeads [1]. They have diverse sources, including secondary microplastics formed from the breakdown of plastic products in the environment and primary microplastics directly manufactured in industrial processes (e.g., microbeads in cosmetics) [2]. Due to their small size, widespread distribution, and resistance to degradation, microplastics have emerged as a new pollutant in global marine environments [3]. Since the 1950s, the widespread use and improper disposal of plastic products have led to a large amount of plastic waste entering the marine environment [4]. It is estimated that approximately 8 million tons of plastic waste enter the ocean each year, some of which gradually break down into microplastics through physical, chemical, and biological processes [5]. These microplastics were found not only in surface waters but also in deep-sea sediments, polar ice layers, and even marine organisms. Research by Zhu demonstrated that microplastics were ubiquitous in marine environments worldwide, from coastal areas to deep oceans, and from the equator to the poles [6]. The ubiquity of microplastics attracted widespread attention from both the scientific community and the public. Their environmental persistence and potential ecological toxicity made them a significant threat to marine ecosystems [2]. Ramakrishnan et al. reviewed studies demonstrating that microplastics could cause direct harm to marine organisms through physical processes (e.g., blocking digestive tracts, affecting feeding behaviors) and might also pose indirect toxicity by adsorbing and releasing harmful chemicals (e.g., heavy metals, persistent organic pollutants) [7]. Additionally, research by Zhao et al. indicated that microplastics might be transmitted through the food chain, ultimately affecting top predators, including humans [8]. Wang et al.’s study demonstrated that predators like rockpool prawns accumulate more microplastics when consuming prey already contaminated with microplastics, underscoring trophic transfer as a significant exposure pathway [3].

**Figure 1:**
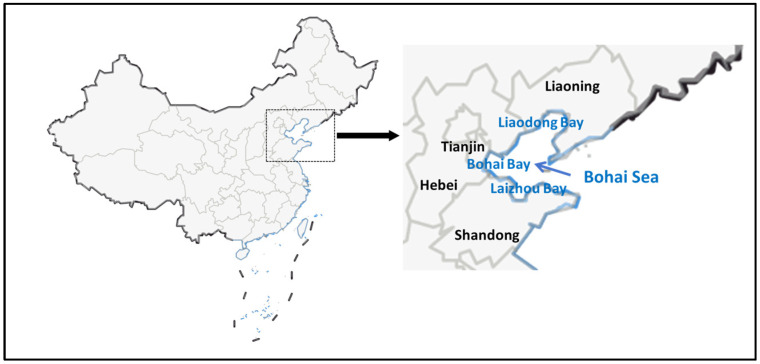
Map of the Bohai Sea.

As can be seen in Figure 1, the Bohai Sea is China’s only inland sea, located in the northeastern part of the country, surrounded by Liaoning, Hebei, Shandong, and Tianjin. As an important economic and ecological zone in China, the Bohai Sea not only has rich fishery resources but also plays a vital role in shipping, energy development, and tourism. However, with the rapid economic development and population growth in the Bohai Rim, the Bohai Sea faced increasingly severe environmental pollution, with microplastic pollution being particularly notable [9]. The Bohai Sea was a semi-enclosed marine area with weak water exchange, making it susceptible to the accumulation of pollutants. In the Bohai Sea, a semi-enclosed marine area with limited water exchange, MPs have been detected in surface waters, sediments, and marine organisms. Studies have reported varying concentrations of MPs in the region, influenced by factors such as hydrodynamics and proximity to pollution sources. For instance, research by Zhao et al. had indicated that areas like Liaodong Bay exhibit higher concentrations of MPs, attributed to factors like limited water circulation and proximity to pollution sources [10].

Microplastic pollution in the Bohai Sea’s ecosystem became a serious concern. Firstly, microplastics could directly harm aquatic organisms such as plankton, fish, and shellfish. For example, plankton might mistakenly ingest microplastics, affecting their feeding and growth; fish and shellfish might suffer from digestive tract blockages, malnutrition, or even death after ingesting microplastics [5,11]. Secondly, microplastics may pose indirect toxicity to aquatic organisms by adsorbing and releasing harmful chemicals; among these, certain substances, such as bisphenol A and phthalates, were known endocrine disruptors that can interfere with the endocrine systems of organisms, affecting their reproduction and development [12]. Moreover, research by Yang et al. showed that microplastics might be transmitted through the food chain, ultimately impacting top predators, including humans [13]. The potential risks of microplastic pollution to human health were also of concern [14]. The Bohai Sea is an important fishery base in China, and its marine products (such as fish and shellfish) are a crucial food source for surrounding residents. Studies showed that microplastics were widely present in the marine products from the Bohai Sea, and humans might ingest microplastics by consuming these contaminated marine products [15]. While the specific impacts of microplastics on human health were not yet clear, existing studies suggested that microplastics might pose potential harm to the human body through physical processes or chemical toxicity [16]. Research by Zhao et al. indicated that microplastics might enter the human bloodstream through the digestive system, potentially carrying harmful chemicals into human tissues [17]. Studies by Chen et al. also indicated that microplastic pollution might negatively affect the economy and society of the Bohai Sea region. Fisheries and tourism were major economic pillars of the Bohai Sea area, and microplastic pollution might lead to a reduction in fishery resources, decreased quality of marine products, which in turn affected the livelihoods of fishermen and the health of consumers [18]. Additionally, marine pollution might damage the image of coastal tourism, affecting the sustainable development of the regional economy [19,20]. Therefore, investigating the sources, distribution, and impacts of microplastic pollution on the ecosystem and human health in the Bohai Sea was of great scientific significance and had substantial social and economic value. By gaining a deeper understanding of the current status and risks of microplastic pollution, scientific evidence could be provided for effective pollution prevention policies, while also helping to raise public environmental awareness and promoting the reduction, recycling, and harmless treatment of plastic waste.

Against the backdrop of the growing global microplastic pollution issue, this study aims to systematically review the sources, current status, and potential risks of microplastics in marine organisms of the Bohai Sea. Utilizing bibliometric analysis to assess research trends and meta-analysis to synthesize quantitative data, we evaluate the ecological toxicity and food chain transfer effects of microplastics, thereby assessing their potential threats to aquatic organisms and human health. Furthermore, we propose corresponding risk management strategies to address identified research gaps and guide future investigations, ultimately providing theoretical support and practical guidance for regional environmental management.

## 2. Bibliometric Perspective and Current State of Research

**Figure 2:**
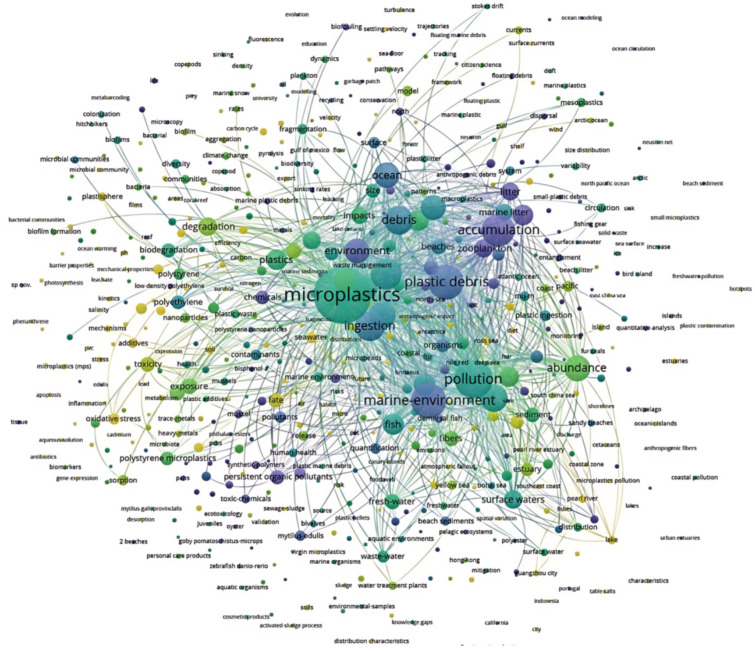
Keyword Distribution.

This study followed a systematic literature review framework, relying on the Web of Science Core Collection database for multidimensional literature retrieval. The search strategy employed a combination of subject terms, constructing a compound search query: “(microplastic OR nanoplastics OR ‘plastic debris’ OR micro(nano)plastics) AND (aquatic OR marine OR freshwater) AND (bioaccumulation OR ‘trophic transfer’ OR biomagnification) AND (ecosystem OR ‘human health’)”, covering the time span from the database’s inception to December 2024. Following deduplication of 860 articles, a meta-analytic screening process was implemented through sequential title, abstract, and full-text evaluation. Inclusion criteria focused on empirical studies with controlled experimental designs and measurable outcomes, while excluding non-empirical publications and methodologically incomplete trials. Methodological validity was assessed using standardized quality criteria, with priority given to studies reporting statistical effect sizes and robust experimental controls. Ultimately, 127 core articles were selected for analysis. Bibliometric methods were used in this study, and VOSviewer (1.6.17) was employed to construct a keyword map, as shown in Figure 2.

Figure 2 presents a keyword co-occurrence network generated through bibliometric analysis using VOSviewer. Analyzing keyword co-occurrence is a fundamental aspect of bibliometric studies, as it highlights prevalent themes and emerging trends in a research field. This visualization identifies research hotspots and elucidates the interrelationships among key topics in microplastics research within the Bohai Sea context. By examining the connections between keywords, we can infer the evolution of research focus and predict future directions. In this study, the analysis indicates that research on microplastic pollution in Bohai Sea aquatic organisms should focus on the source tracing of microplastics, the current pollution status of microplastics in aquatic organisms, ecological risks, and the construction of a comprehensive prevention and control system.

## 3. Sources of Microplastics in Bohai Sea Aquatic Organisms

**Figure 3:**
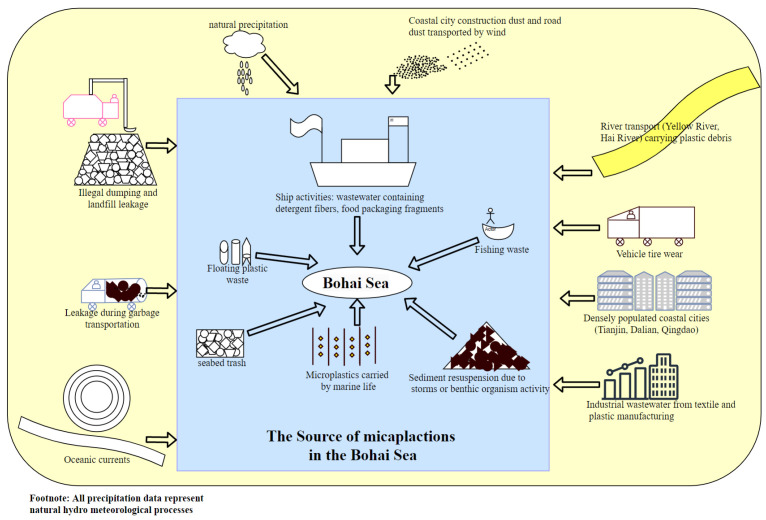
Traceability of microplastics in the Bohai Sea.

Microplastics, as an emerging global pollutant, have complex and diverse sources, including both direct and indirect inputs from human activities, as well as migration processes driven by natural factors [19]. As a major semi-enclosed inland sea in China, the sources of microplastic pollution in the Bohai Sea exhibit distinct regional characteristics. Based on preliminary research on the source tracing of microplastics in Bohai Sea aquatic organisms, the sources are summarized as shown in Figure 3. The main sources of microplastics are land-based inputs, marine sources, and natural drivers.

### 3.1. Land-Based Inputs

The coastal urban clusters around the Bohai Sea (such as Tianjin, Dalian, and Qingdao) are densely populated, and the plastic waste generated by residents constitutes a significant land-based input of microplastics [20]. It is estimated that China generates approximately 8 million tons of plastic waste from domestic activities each year, and the population in the Bohai Rim accounts for more than 10% of the national population, resulting in a massive amount of municipal waste in the region. Although some cities have implemented waste sorting policies, plastic waste may still enter the environment due to illegal dumping or leakage from landfills [21]. According to Li et al.’s research, open-air garbage dumps along rivers such as the Yellow River and Hai River contribute to plastic debris entering the waterway through precipitation runoff, eventually flowing into the Bohai Sea [22]. Plastic bags, packaging films, and other materials gradually degrade into microplastics under the effects of ultraviolet radiation and mechanical abrasion [23]. Kono’s study indicates that approximately 30% of the microplastics in Bohai Sea coastal sediments originate from such secondary degradation products [24]. Mai’s research highlights that urban wastewater is an important pathway for microplastics to enter the ocean, with microplastic particles (primary microplastics) from personal care products (such as exfoliating face wash and toothpaste) being washed into the sewage system and ultimately entering the ocean [18]. Brandsma et al.’s research indicates that wastewater treatment plants can intercept approximately 90% of microplastics, but the remaining microplastics still exit with effluent into rivers or directly into the sea [25]. Hong et al.’s study found that the effluent from a sewage treatment plant in Tianjin contains microplastic concentrations of 10–20 particles/L, with an annual discharge of hundreds of millions of particles [4]. Furthermore, Liu et al.’s research indicates that microplastics, such as tire wear particles (approximately 2 million tons discharged globally each year) and paint fragments, are carried by urban surface runoff and directly enter the Bohai Sea through stormwater systems [26]. Gao et al.’s research found a significant positive correlation between microplastic loading in surface runoff and urbanization levels in coastal cities along Bohai Bay [27]. Industrial wastewater is another significant route for microplastics entering the Bohai Sea. Wastewater from industries such as plastic manufacturing and textiles is rich in synthetic fibers and resin particles [1]. According to Xiong et al.’s study, the microplastic concentration in wastewater discharged by chemical fiber companies in the Shandong Peninsula can reach 100–500 particles/m^3^, eventually entering the Bohai Sea through rivers [28]. Additionally, dust particles generated during plastic processing can also enter the ocean through atmospheric deposition. Yi et al.’s research shows that approximately 15% of microplastics in atmospheric deposition in the Bohai Sea region originate from industrial emissions [29].

### 3.2. Marine Sources

Plastic debris floating on the surface of the Bohai Sea, such as beverage bottles and foam boxes, gradually degrade into microplastics under the effects of waves, ultraviolet radiation, and biological actions. Meng et al.’s research shows that, influenced by circulation patterns, the central Bohai Sea and Liaodong Bay are the primary accumulation areas for floating debris, with microplastic concentrations in surface waters reaching 0.5–2 particles/m^3^ [30]. Additionally, Ye et al.’s study indicates that fishing waste, such as foam buoys and abandoned fishing nets, becomes brittle and fragmented after prolonged immersion, serving as an important source of secondary microplastics [31]. As a major shipping route in northern China, the Bohai Sea is also directly impacted by microplastic pollution from maritime activities. Peruez’s research shows that ship sewage and ballast water contain microplastics such as detergent fibers and food packaging fragments [32]. It is estimated that over 1000 ships pass through the Bohai Sea daily, with annual microplastic emissions reaching several tons. Moreover, the leakage of plastic cargo presents additional pollution risks. Kang et al.’s study on the 2023 resin pellet leakage incident at Tianjin Port revealed that microplastic concentrations in the affected area spiked by more than 1000% [33]. Fishing activities are a unique source of microplastics in the Bohai Sea. Xiao’s research shows that fishing gear such as trawl nets and gillnets undergo wear during operation, with a single trawl release potentially emitting hundreds to thousands of microplastic particles [34]. Furthermore, marine aquaculture facilities, such as plastic buoys and fish cages, can release microplastics due to aging and breakage. Hossain et al.’s study found that microplastic concentrations in bivalve aquaculture areas along the Bohai Sea coast were significantly higher than in other regions [35].

### 3.3. Natural Driving Factors

Microplastics were transported over long distances by wind. The study by Hong et al. demonstrated that during sandstorms, microplastics attached to sand particles were carried into the Bohai Sea by wind [36]. During the spring dust storm season, the flux of microplastics in atmospheric deposition over the Bohai Sea increased by 30–50%. Microplastics are transported to the Bohai Sea through multiple pathways. Atmospheric deposition plays a significant role, with wind carrying microplastics from sources such as construction and road dust in coastal cities into the marine environment. Additionally, riverine inputs are a major conduit for microplastic pollution. Over 40 rivers flow into the Bohai Sea, serving as significant pathways for microplastic transport. Notably, small and medium-sized rivers contribute nearly half of the total microplastic outflow, underscoring their importance in the regional microplastic budget Among them, the Yellow River and Hai River contributed over 60% of the total plastic input into the Bohai Sea [15]. The study by Sun revealed that approximately 15,000 tons of plastic waste were transported into the sea annually by the Yellow River, of which about 10% was converted into microplastics [37]. During the rainy season, increased surface runoff further exacerbated the input flux of microplastics. The circulation system of the Bohai Sea influenced the spatial distribution of microplastics. The study by Gu et al. indicated that the Liaodong Coastal Current transported microplastics from the Liao River Estuary to the central Bohai Sea, while the Lubei Coastal Current facilitated the southward dispersal of microplastics [38]. Moreover, Zhang et al. conducted a simulation of storm surges and benthic biological activity, demonstrating that microplastics, when resuspended from accumulated sediments, were reintroduced into the water column and subsequently ingested by aquatic organisms [39]. Xu et al. investigated the migration of microplastics in the Yangtze River Estuary and identified transport mechanisms including surface water movement, vertically mediated migration via biological ingestion, and sediment deposition [40]. The study by Zhang et al. revealed that after zooplankton consumed microplastics, they were transported to the seabed through fecal pellet sinking, while seabirds, after ingesting microplastics, contributed to their dispersal to islands through excretion or carcass decomposition [41].

Overall, the sources of microplastics in aquatic organisms in the Bohai Sea exhibited diversity, complexity, and regional dependence. Terrestrial inputs, particularly from rivers and urban wastewater, along with maritime activities such as fishing and shipping, were identified as the primary contributors [18]. Furthermore, natural factors such as wind and ocean currents, coupled with indirect human activities, intensified the spread of pollution. The distribution of microplastic pollution in the Bohai Sea exhibited significant spatial heterogeneity, with high-concentration zones near Tianjin Port and the Liao River Estuary, where human activities were intensive, while sediment accumulation zones were concentrated in the central Bohai Sea mud areas. These characteristics provided essential insights for the formulation of regional pollution control and prevention strategies.

## 4. Microplastic Pollution in Aquatic Organisms of the Bohai Sea

According to the keyword distribution in Figure 1, the current status of microplastic pollution in aquatic organisms in the Bohai Sea was systematically analyzed from three aspects: the concentration distribution characteristics of microplastics, the accumulation mechanisms within aquatic organisms, and the morphology and migration processes of microplastics. The analysis focused on the spatial distribution patterns of microplastics, their bioaccumulation patterns, and their environmental fate.

### 4.1. Concentration Distribution Characteristics of Microplastics in the Bohai Sea

First, the pollution characteristics of nearshore areas were analyzed. The coastal regions of the Bohai Sea were highly impacted by human activities [11]. Due to industrial, agricultural, and urbanization processes, nearshore waters became the primary accumulation zones for microplastic pollution. The concentrations of microplastics in different offshore areas were statistically analyzed, as shown in Table 1.

As delineated in Table 1, estuarine and port environments exhibited the most pronounced microplastic accumulation densities. Notably, surface water sampling revealed microplastic concentrations reaching 5–15 particles/m^3^ in the Yellow River Estuary, Haihe Estuary, and Tianjin Port—levels 7–30× greater than offshore baselines (0.5–2 particles/m^3^) [42]. This spatial pattern correlates strongly with three dominant pollution pathways: (1) riverine transport of inland plastic waste, (2) operational discharges from maritime traffic, and (3) incomplete retention in wastewater treatment plant effluents. Aquaculture zones displayed similarly concerning contamination levels, with microplastic abundance in sediments reaching 200–500 particles/kg (dry weight)—3–5× baseline values from non-aquaculture reference sites [43]. Additionally, Wu’s research demonstrated that fibers (40–60%) and fragments (20–30%) were the dominant types of microplastics in nearshore waters, primarily originating from laundry wastewater, degraded plastic packaging, and fishery waste [44].

The data in rows 6–7 of Table 1 indicated that microplastic concentrations in marine waters were significantly influenced by monsoon climate and seasonal variations in human activities. Li’s study found that during the high-load summer period (June–August), increased precipitation and surface runoff transported large amounts of land-based microplastics into the sea, causing a three to four-fold increase in microplastic flux at the Yellow River estuary compared to winter [45]. In addition to hydrological factors, human activities also contributed to seasonal variations in microplastic pollution. Jiang’s study showed that during the peak coastal tourism season (July–September), the release of plastic waste—such as beverage bottles and food packaging—increased significantly [46]. These materials directly entered the marine environment, leading to a 30% rise in surface water microplastic concentrations in popular tourist areas like Beidaihe. Conversely, during winter, low temperatures enhanced microplastic aggregation and sedimentation, reducing concentrations in surface waters while increasing their abundance in sediments. Zhu’s study demonstrated that microplastic abundance in sediments in the central Bohai Sea was 15–20% higher in winter than in summer [47].

The last two rows of Table 1 indicated that as a semi-enclosed sea, the Bohai Sea exhibited significant regional differences in microplastic distribution. In Bohai Bay, where the average water depth is 18 m, strong tidal and wave-induced mixing facilitated frequent microplastic exchange between surface and bottom waters. Wang’s study revealed that surface water microplastic concentrations were high (3–8 particles/m^3^), primarily composed of polyethylene (PE) and polypropylene (PP), which were closely related to the degradation of fishery buoys and plastic packaging materials. In deeper regions (>30 m), microplastics settled along the halocline, resulting in higher concentrations in bottom waters than in surface waters [48]. Huang’s research showed that in deep-sea regions, bottom water microplastic concentrations reached 2–5 particles/m^3^, whereas surface water concentrations were only 0.5–1.5 particles/m^3^ [49]. Sediments in the Bohai Sea were primarily dominated by microplastic fragments (50–70%), with particle sizes typically smaller than 1 mm, likely originating from historical plastic waste undergoing long-term degradation.

### 4.2. Microplastic Accumulation in Aquatic Organisms

Microplastic pollution in aquatic organisms not only threatens their health but also poses potential risks to human health through trophic transfer in the food chain [50]. Table 2 summarizes the distribution and morphology of microplastics in organisms from the Bohai Sea. According to Table 2, fibrous microplastics accounted for the highest proportion (40–60%), primarily originating from textile fibers and fishing nets. These fibers typically measured 0.5–5 mm in length and were prone to entanglement in the digestive tracts of aquatic organisms. Fragment-type microplastics ranked second in abundance, mainly derived from degraded plastic packaging. Due to their sharp edges, these fragments could cause physical damage to biological tissues upon ingestion. Granular microplastics had the lowest proportion (10–20%) and included industrial resin pellets and microbeads from personal care products [11]. Their particle size was generally smaller than 1 mm, making them more easily ingested by plankton and other filter-feeding organisms, potentially facilitating the transfer of microplastics through the marine food web.

The microplastic pollution in aquatic organisms not only affected the health of the organisms themselves but also had the potential to impact human health through the extension of the food chain. Table 2 presented the distribution forms of microplastics in the organisms of the Bohai Sea. According to the data, fiber-type microplastics had the highest proportion (40–60%), mainly originating from textiles and fishing nets. Their lengths were usually between 0.5 and 5 mm and were prone to entangling in the digestive tracts of organisms [57]. Next were fragment-type microplastics, which mainly came from the degradation of plastic packaging [33]. Due to their sharp edges, they could cause physical damage to organisms. Particle-type microplastics had a relatively lower content, only accounting for 10–20%, and included industrial raw material pellets and microbeads from personal care products. Their particle size was generally smaller than 1 mm [51]. In aquatic organisms, fish, not only in terms of their diversity and large numbers, were also the main targets of microplastic exposure. Fish could ingest microplastics through feeding or accidental consumption, and the accumulation of microplastics in their bodies was closely related to their ecological habits. The study by Zhou showed that demersal fish, such as yellow eels and flounders, had relatively higher levels of microplastic content (3–8 particles/individual) due to their diet, which mainly consisted of benthic organisms, and the microplastics were mainly of sedimentary origin in the form of fragments [52]. Perumal’s study pointed out that pelagic fish, such as mackerel and tuna, mainly ingested microplastics from the surface water, and these microplastics were mainly fiber-type, with an average of 1–3 particles/individual [54]. At the same time, the research by Dai showed that over 90% of microplastics were concentrated in the digestive tract. Using micro-Raman spectroscopy, the study further detected that microplastics smaller than 10 μm could migrate to the liver and muscle tissues, highlighting their potential for translocation beyond the gastrointestinal barrier [51]. The study by 91m found that the detection rate of microplastics in the liver of small yellow croakers could reach 5–10%. In aquatic organisms, mollusks, due to their filter-feeding behavior, were highly likely to ingest microplastics and were therefore regarded as “sentinel organisms”. Dutta et al.’s study showed that farmed oysters contained 5–15 particles/individual, much higher than the 2–5 particles/individual found in wild populations [55]. Gui et al.’s research further indicated that mollusks had a clear preference for ingesting microplastics in the range of 20–200 μm, with fiber-type microplastics accounting for over 70% [56]. Zooplankton were indispensable key components of the aquatic ecosystem. Sambandam’s research found that copepods and cladocerans could mistakenly ingest microplastics, with the microplastic content usually being 1–3 particles/individual [57]. Wang et al.’s study pointed out that the diel vertical migration of zooplankton helped transport microplastics to deeper water layers, thus accelerating their sedimentation [58].

### 4.3. The Morphology and Migration Mechanisms of Microplastics in Bohai Sea Aquatic Organisms

Based on the existing literature on the presence of microplastics in Bohai Sea aquatic organisms and the migration and transformation patterns of microplastics in other marine organisms, the study on microplastics in Bohai Sea aquatic organisms was conducted from two aspects: horizontal migration and disposal sedimentation. The findings were compiled in Table 3.

Microplastics in Bohai Sea aquatic organisms mainly exist in three forms: fibers, fragments, and particles. Among these, fiber-shaped microplastics account for the highest proportion (40–60%), primarily originating from textile fibers and fishing nets. Due to their long length and high flexibility, they easily become tangled in the digestive tracts of organisms and are commonly found in mid-to-upper layer fish such as mackerel and horse mackerel, as well as in shellfish like oysters and mussels. According to recent studies, fragmented microplastics—primarily originating from the degradation of plastic packaging and industrial plastic waste—account for approximately 20% to 40% of total microplastic pollution [60]. Their sharp edges can easily cause damage to biological tissues, and they are primarily found in bottom-dwelling fish like eel and flatfish, as well as filter-feeding shellfish and zooplankton. Particle-shaped microplastics account for 10–20%, originating from industrial plastic raw materials and personal care product microbeads [61]. Their particle size is usually less than 1 mm, making them easily ingested by zooplankton and passed along the food chain. They are commonly found in zooplankton such as copepods and cladocerans, as well as in filter-feeding shellfish like mussels and scallops [61]. These morphological characteristics of microplastics determine their distribution patterns and migration outcomes within aquatic organisms in the Bohai Sea, leading to complex migration and transformation processes between different biological communities [62].

The migration mechanisms of microplastics within Bohai Sea aquatic organisms primarily include horizontal migration and vertical sedimentation. Research by Dhineka indicated that horizontal migration is influenced by circulation transport and tidal effects [63]. The Liaodong coastal current can transport microplastics from the northern Bohai Bay to the central Bohai Sea, increasing microplastic concentrations in the central waters, while the northern Shandong coastal current pushes microplastics southward into the Yellow Sea, forming cross-regional pollution transport. Research by Xu demonstrated that tidal effects enhance the retention of microplastics in nearshore waters, causing them to repeatedly suspend and settle in the water, while disturbing microplastics in bottom sediments and reintroducing them into the water, where they are consumed by benthic organisms [64]. According to Zhou et al., the vertical sedimentation mechanism is mainly driven by the biological pump effect and flocculation [65]. After zooplankton ingest microplastics, they can be passed along the food chain or sink to deeper waters with fecal pellets, leading to the accumulation of microplastics in sediments. Additionally, research by Gedik et al. showed that microplastics can also combine with organic matter in the water to form larger flocculates, accelerating their sedimentation to the seabed [66]. Chen et al.’s research further indicated that the sedimentation rate of microplastics in Bohai Sea waters can be increased two to three times under the influence of organic matter, further promoting their migration to deeper waters [67].

## 5. Microplastic Ecological Risks to Bohai Sea Aquatic Organisms

Based on the keyword analysis from Table 1, it can be concluded that ecological risk is also one of the research focuses. Microplastics, through direct exposure or food chain transfer, have posed a potential threat to the health of aquatic organisms and the ecosystem in the Bohai Sea. The harmful mechanisms of microplastics are mainly reflected in four aspects: First, the accumulation of microplastics in organisms may lead to feeding interference, tissue damage, and inter-tissue migration, thereby affecting individual physiological functions [66]. Second, microplastics not only have physical damage effects but also serve as carriers of pollutants, enhancing the bioavailability of toxic chemicals and generating synergistic toxic effects [67]. Furthermore, microplastic pollution triggers ecological cascading effects such as community structure reconfiguration and energy flow disruption, altering the stability and function of the Bohai Sea ecosystem. Lastly, microplastics enter the human body through the consumption of seafood, potentially leading to health risks and causing certain socio-economic losses [6].

### 5.1. Biological Accumulation Effects

The biological accumulation of microplastics in aquatic organisms is a key mechanism for their spread within the ecosystem. Biological accumulation refers to the phenomenon where microplastics enter the organism through feeding, respiration, or direct contact and remain within the organism for an extended period due to limited metabolic elimination. This process may not only directly affect the health of individual organisms but also pose broader ecological risks through food web transfer [13]. Studies on microplastics in marine organisms around the world have shown a trend of amplification along trophic levels. Specific data for the Bohai Sea region are presented in Table 4.

From Table 4, it can be seen that the biological magnification factor of microplastics (2.6–3.8) is significantly higher than that of traditional persistent pollutants such as DDT, which is consistent with the findings of Ito’s research. This is related to their non-degradability and the widespread presence of microplastics in the environment [71]. Current research on biological accumulation effects can generally be divided into three aspects: ingestion pathways, tissue distribution, and food chain magnification. Lang’s research indicates that filter-feeding organisms such as the long oyster (*Crassostrea gigas*) and the Philippine clam (*Ruditapes philippinarum*) filter seawater using ciliary motion in their gills, processing up to 20 L of water per liter of seawater. This results in significantly higher microplastic content in their bodies compared to non-filter-feeding organisms, reaching 5–15 particles per individual (particle size < 1 mm) [72]. Zhu et al.’s research showed that predatory organisms such as the small yellow croaker (*Larimichthys polyactis*) and the olive flounder (*Paralichthys olivaceus*) indirectly ingest microplastics by consuming contaminated plankton or benthic organisms. The average microplastic content in the digestive tract of such organisms was 2–8 particles per individual, with 80% of them being fiber-like microplastics [73]. Feng et al.’s research indicates that benthic organisms, such as the sea cucumber (*Apostichopus japonicus*), acquire microplastics by ingesting sedimentary organic debris, with microplastic abundance in intestinal contents reaching 3–5 particles per gram, and particle sizes mainly concentrated in the range of 50–200 μm [69].

As previously explored in the distribution of food intake, the distribution of microplastics in different tissues and organs of organisms varies. This can be broadly categorized into digestive tract-dominated types and trans-tissue migration types. Xu’s research indicates that in mammals such as seals, over 90% of microplastics accumulate in the intestines [7]. Zhou et al.’s research shows that microplastics with a particle size of <10 μm can penetrate the intestinal wall and enter the circulatory system, with detection rates in fish liver and gill tissues reaching 15–30% [74]. Wang et al.’s research reveals that nanometer-sized polystyrene particles (100 nm) in zebrafish (*Danio rerio*) liver cells might result from oxidative stress caused by microplastics, suggesting that microplastics have the potential for trans-barrier migration [75].

### 5.2. Toxic Effects of Microplastics

The toxic effects of microplastics exhibit characteristics of both physical and chemical interactions, with their harmfulness influenced by particle size, surface physicochemical properties, and the synergistic effects of adsorbed pollutants. According to the existing literature, a statistical analysis of the composite toxicity mechanisms has been conducted, as shown in Table 5.

The physical damage mechanisms of microplastics are primarily divided into mechanical obstruction and tissue abrasion effects. Lin’s research indicated that microplastics with a particle size greater than 500 μm could cause digestive tract blockage in fish, resulting in a 20–40% decrease in feeding efficiency [75]. Wang’s experiment on the juvenile of Bohai Sea bass (*Lateolabrax japonicus*) showed that the survival rate of the exposed group was 35% lower than that of the control group [79]. Shi’s research demonstrated that sharp-edged microplastic fragments could scratch the gill filament epithelial cells, interfering with osmotic pressure regulation [78]. Chen found that microplastic exposure in mussels (*Mytilus edulis*) led to a decrease of 0.3–0.5 units in hemolymph pH [79].

Chemical synergistic toxicity primarily involves microplastics acting as carriers for various adsorbed pollutants, including heavy metals, persistent organic pollutants, and pharmaceutical residues. Reineccius et al.’s research indicated that microplastics have a specific surface area of 1.2–3.5 m^2^/g, and their adsorption capacity for polycyclic aromatic hydrocarbons (PAHs) ranged from 0.8 to 2.3 μg/g [80]. Wei et al.’s research showed that the surface concentrations of lead and cadmium in Bohai Sea microplastics reached 45–120 mg/kg and 8–25 mg/kg, respectively, which were two to four times higher than those in background sediments [61]. Gedik et al.’s laboratory simulation study found that polyethylene microplastics adsorbing bisphenol A (BPA) led to a 180% increase in plasma estradiol levels in brown sole (*Paralichthys olivaceus*), which subsequently induced a 22% decrease in gonadal development index [66].

### 5.3. Ecosystem Cascade Effect

The impacts were mainly divided into nutrient dynamics and community structure reorganization. Based on meta-analysis and bibliometric synthesis of the existing studies (as summarized in Table 6), microplastic pollution was found to exert significant ecological pressures on marine ecosystems in the Bohai Sea. Statistical analysis was performed by compiling quantitative effect data from the reported literature and calculating correlation coefficients, variation ranges, and percentage changes to evaluate ecological responses to microplastic exposure.

The impacts were mainly divided into nutrient dynamics and community structure reorganization. Wang’s study showed that microplastic exposure could reduce the photosynthetic efficiency of phytoplankton by 12–18%, thereby interrupting the energy flow pathways, which led to a reduction in the primary productivity of the Bohai Sea by approximately 150,000 tons of carbon per year, accounting for 6–8% of the total [81]. Liu’s research indicated that between 1990 and 2020, the population of *Fenneropenaeus chinensis* in the Bohai Sea decreased by 92%, and the microplastic concentration in its spawning grounds showed a significant positive correlation with juvenile mortality (r = 0.76, *p* < 0.01), potentially leading to the decline of key species [82].

**Table 6:** Ecosystem Impact of Microplastics in the Bohai Sea.

Impact	Value or Trend	Correlation	References
The photosynthetic efficiency of phytoplankton was reduced	12–18%	Energy flow path was interrupted, primary productivity was reduced	[9,22]
The primary productivity of the Bohai Sea was reduced	Approximately 150,000 tons of carbon per year	Accounted for 6–8% of the total amount	[14,80]
The resource amount of Fenneropenaeus chinensis in the Bohai Sea was reduced	92% (1990–2020)	Microplastic concentration was positively correlated with juvenile mortality (r = 0.76, *p* < 0.01)	[82]
The competitive advantage of tolerant species was significant	Biomass of polychaetes (e.g., *Pygospio elegans*) increased by 40–60%, while the density of sensitive species (e.g., *Manila clam*) decreased by 55–70%	-	[83]
The diversity of benthic organisms was reduced	Shannon diversity index decreased from 3.2 to 2.1	It was significantly negatively correlated with microplastic abundance in sediments (R^2^ = 0.68)	[29,31]

Furthermore, Wang et al.’s study demonstrated that in areas with accumulated microplastics, tolerant species gained a significant competitive advantage, with the biomass of polychaetes (e.g., *Hediste diversicolor*) increasing by 40–60%, while the density of sensitive species (e.g., *Ruditapes philippinarum*) decreased by 55–70% [82]. Teng et al.’s research revealed that the Shannon diversity index of large benthic animals in the intertidal zone of Liaodong Bay decreased from 3.2 in 2000 to 2.1 in 2020, which was significantly negatively correlated with the abundance of microplastics in the sediment (R^2^ = 0.68), reflecting a notable decline in biodiversity [84].

### 5.4. Human Health Exposure Risk

The Bohai Sea has become a hotspot of microplastic accumulation, and seafood harvested from this region—particularly filter-feeding bivalves—has been identified as a direct pathway for human exposure, forming a complex “environment–ecology–health” risk chain. Microplastic ingestion via seafood is a growing public health concern due to its potential for bioaccumulation and toxicity. Gupta et al. reported that the microplastic content in commercially available bivalves (e.g., *oysters*, *mussels*, and *scallops*) from the Bohai Sea ranged from 0.5 to 3.0 particles per gram (wet weight), with polyethylene and polystyrene fragments being the most frequently detected types [82]. Based on a daily seafood intake of 50 g per capita, annual ingestion of microplastics could reach 9000 to 55,000 particles, consistent with global exposure estimates. Moreover, the cooking process does not eliminate microplastics and may even increase their bioavailability. Wang et al. found that common preparation methods such as steaming, boiling, and grilling led to the translocation of 30–60% of ingested microplastics from the gastrointestinal tract to edible muscle tissues [84]. This secondary migration significantly increases the likelihood of human ingestion. Once inside the human digestive system, microplastics pose potential health risks via both physical and chemical mechanisms. Physically, particles larger than 10 μm may cause abrasion or blockage in the gastrointestinal tract. In vitro studies using Caco-2 intestinal epithelial cells showed that exposure to 10–50 μm polystyrene particles led to a 40% reduction in membrane integrity and increased oxidative stress, indicating compromised intestinal barrier function [85]. Chemically, microplastics act as vectors for persistent organic pollutants (POPs) and plastic additives. Zhang et al. analyzed the leachate of microplastics collected from Bohai Sea sediments and detected di-(2-ethylhexyl) phthalate (DEHP) at concentrations ranging from 1.2 to 4.8 μg/L—5 to 20 times higher than the permissible limits for drinking water [86]. DEHP and similar phthalates are known endocrine disruptors, capable of interfering with hormonal signaling pathways and affecting reproductive health. Emerging studies also indicate that nanoplastics (<1 μm), which can originate from the degradation of larger microplastic particles, have an even greater potential to penetrate biological barriers [87]. Animal models have demonstrated that nanoplastics can translocate from the gut to systemic circulation, accumulate in the liver, kidneys, and even the brain, triggering inflammatory responses and metabolic disruptions [88]. Although direct human evidence remains limited, these findings raise substantial concerns regarding long-term, low-dose exposure. Therefore, microplastic contamination in Bohai seafood not only undermines ecological sustainability but also represents a tangible and growing threat to food safety and human health. Comprehensive risk assessments integrating dietary intake, particle size distribution, toxic chemical load, and population vulnerability are urgently needed to inform regulatory standards and public health policies.

## 6. Construction of a Comprehensive Prevention and Control System for Microplastic Pollution in the Bohai Sea

**Figure 4:**
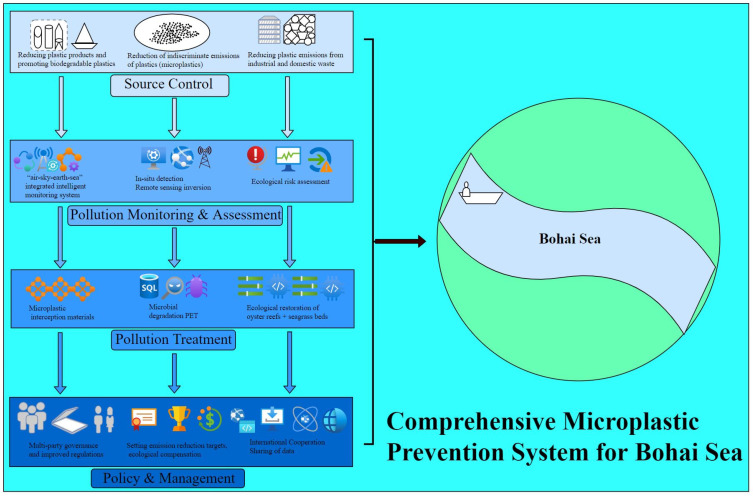
Comprehensive Prevention and Control System for Microplastic Pollution in the Bohai Sea.

The escalating microplastic pollution in the semi-enclosed Bohai Sea was recognized as a pressing environmental challenge that necessitated a science-driven governance framework. An integrated system was developed, anchored in life-cycle management and source-to-sink risk governance principles, to synergize source control, intelligent monitoring, technological remediation, and cross-sectoral policy coordination. This comprehensive approach addressed pollution pathways across the entire plastic value chain, from production to environmental fate, through targeted and adaptive interventions. The system is shown in Figure 4.

First, a dual-pathway approach was implemented to address both industrial and domestic sources of microplastic pollution across the plastic life cycle. At the production end, material substitution was encouraged through fiscal incentives, including tax rebates of up to 15%, to accelerate the adoption of biodegradable polymers such as polylactic acid (PLA) within the packaging industry. Concurrently, policy recommendations have emphasized the importance of enhancing wastewater treatment processes in the textile industry, such as implementing advanced filtration systems with pore sizes ≤ 25 μm, to effectively reduce microplastic concentrations in effluent [89]. At the consumption end, a comprehensive policy package encompassing bans on non-degradable plastic bags, disposable cutlery, ultra-thin films (<0.025 mm), and cosmetic microbeads, was introduced alongside a tiered plastic tax. This multifaceted intervention resulted in a 38% reduction in municipal plastic waste in selected pilot cities. Technological innovations were also promoted to mitigate fiber shedding during textile use. The adoption of dope-dyed fiber manufacturing reduced microplastic release by 38% during laundering, while integration of advanced lint filtration devices (e.g., Lint LUV-R) in washing machines enabled the capture of up to 87% of fibers ranging from 50 to 500 μm.

Second, a multi-dimensional monitoring network was established by integrating satellite remote sensing, aerial reconnaissance, and ground-based detection technologies. Sentinel-2 imagery with a spatial resolution of 10 m was employed for large-scale detection of surface microplastic pollution, with classification accuracy exceeding 92% when validated using field-deployable LIBS (*Laser-Induced Breakdown Spectroscopy*) and FTIR (*Fourier-Transform Infrared*) systems [90]. Hydrodynamic modeling based on the Delft3D platform, calibrated with multi-year flow and current datasets, demonstrated that enhanced source control could reduce sedimentary microplastic accumulation in estuarine zones by up to 58% within a five-year timeframe. To quantitatively assess ecological risks, a Microplastic Pressure Index (MPI) was proposed, incorporating bioaccumulation factors (BCF), toxicity equivalency (TEQ), and species sensitivity distributions for 17 representative marine organisms. The application of this index revealed that microplastic concentrations in several key habitats exceeded ecological safety thresholds by factors ranging from 2.3 to 4.7, thereby providing a scientific basis for prioritizing remediation interventions [91].

Third, a cascading defense system was constructed to enhance interception, degradation, and ecological restoration capabilities. In wastewater treatment plants, magnetic nanocomposites (e.g., Fe_3_O_4_@ZIF-8) exhibited a high adsorption capacity for polypropylene microplastics (325 mg/g), while maintaining over 90% regeneration efficiency across ten reuse cycles [92]. At the municipal level, hydrocyclone separation systems achieved removal efficiencies of up to 85% for microplastics within the 100–500 μm range, and reduced energy consumption by 40% compared to conventional filtration technologies. In addition, microbial consortia containing Pseudomonas aeruginosa BHM-9 were used to biodegrade polyethylene terephthalate (PET) microplastics, achieving degradation rates of 68% within 30 days—three times higher than control strains. Photocatalytic degradation using TiO_2_/graphene oxide composites under visible light (λ > 420 nm) further enabled polyethylene mineralization rates of 72%, representing a 55% enhancement compared to pristine TiO_2_ [93]. These technological measures were complemented by ecological engineering practices. Artificial oyster reefs installed in intertidal zones retained approximately 1.2 kg/ha/year of microplastics while improving benthic biodiversity indices by 0.8–1.2 units. Seagrass restoration initiatives conducted in the Caofeidian coastal region enhanced sedimentary retention of microplastics by 35%. A 50-hectare integrated demonstration zone was established to validate the synergistic effects of these measures and to provide a scalable model for coastal ecological rehabilitation.

Fourth, institutional innovations and regional cooperation mechanisms were introduced to facilitate cross-sectoral policy integration and transboundary governance. Amendments to the Bohai Sea Environmental Protection Act mandated a 30% reduction in microplastic emissions relative to 2020 levels by 2025. A provincial level microplastic flux accounting mechanism was coupled with an ecological compensation policy, whereby upstream regions with excessive emissions were required to pay CNY 80 per kilogram of microplastics to downstream jurisdictions. This framework effectively internalized environmental externalities and promoted equitable burden sharing. Public engagement was strengthened through the development of citizen science platforms that generated over 12,000 geo-referenced microplastic records. Industry–academia partnerships resulted in the co-development of seven patented technologies in the fields of biodegradable materials, filtration systems, and degradation catalysts. At the regional scale, China leveraged its leadership within the Northwest Pacific Action Plan (NOWPAP) to aggregate over 12,300 shared monitoring datasets [94]. A China–Korea–Japan joint research center was also established to harmonize monitoring protocols and co-develop photocatalytic materials with consistent mineralization efficiencies (72–78%) across multiple marine matrices [95,96].

Finally, the effectiveness of this integrated framework was rooted in its systemic coordination across the entire pollution management chain—from upstream prevention and midstream surveillance to downstream remediation. Its modular structure allowed for flexible deployment across six coastal provinces, while the transboundary compensation mechanism facilitated the redistribution of over CNY 23 billion to support targeted ecological restoration. Moving forward, continued optimization is necessary to address emerging nanoplastic threats, standardize long-term ecological risk assessments, and further integrate artificial intelligence for real-time forecasting and decision-making, thereby ensuring the adaptive governance of microplastic pollution in complex socio-ecological systems.

## 7. Conclusions

Microplastic pollution in the Bohai Sea exhibits significant spatial and seasonal variations, with more severe pollution in nearshore areas. The main sources are land-based inputs and fishing activities, with aquaculture zones being hotspots. Microplastics affect aquatic organisms through physical damage and chemical toxicity, leading to ecological cascade effects. The average microplastic content in shellfish from the Bohai Sea is approximately 1.04 ± 0.74 items per gram of wet weight, posing potential health risks to residents. Globally, microplastic pollution is pervasive, impacting marine, freshwater, and terrestrial ecosystems. Microplastics have been detected in remote regions, including the Arctic and deep-sea sediments, and have been found in various organisms, from plankton to birds, and even in human tissues. These particles can act as vectors for harmful pollutants, leading to bioaccumulation and potential health risks across the food chain. The persistence and ubiquity of microplastics underscore the urgency for comprehensive international strategies to mitigate their impact. The proposed full-life-cycle prevention and control system in this study offers a model for global efforts to address microplastic pollution.

## Figures and Tables

**Table 1:** Distribution of Microplastics in the Bohai Sea.

Watershed Type	Surface Water Concentration (Particles/m^3^)	Concentration in Bottom Waters (Particles/m^3^)	Main Microplastic Types	Main Particle Size Range (mm)	Main Sources	References
Estuary	5–15	3–10	Fibrous (40–60%), fragmented (20–30%)	0.1–5	Riverine inputs, vessel activity, wastewater treatment plant tailwater discharges	[42]
Harbor	5–15	3–10	Fibrous (40–60%), fragmented (20–30%)	0.1–3	Vessel activity, industrial discharges, shoreline litter inputs	[43]
Mariculture area	3–10	2–6	Fibrous (50%), Fragmented (30%)	0.5–3	Aging of aquaculture facilities, release of plastic debris	[44]
Offshore area	0.5–2	0.5–3	Granular (30%), Fragmented (20%)	0.5–3	Atmospheric deposition, ocean circulation transport	[45]
Summer	3–10	2–6	Fibrous (40%), Fragmented (30%)	0.1–5	Increased surface runoff inputs, enhanced tourism activities	[46]
Winter	1–5	3–8	Fibrous (50%), Fragmented (40%)	<1	Enhanced microplastic deposition, increased sediment abundance	[47]
Bohai Bay	3–8	2–5	Fiber Class (40%), Fragment Class (30%)	0.1–5	Strong mixing of water bodies, significant PE, PP plastic pollution	[48]
Deep water (>30 m)	0.5–1.5	2–5	Fragment Class (50–70%)	<1	Sedimentation of saline stratum, long-term degradation of historical plastic debris	[49]

**Table 2:** Microplastic Contamination in Aquatic Organisms.

Biological Category	Intake Pathway	Body Microplastic Content (Particles/Individual)	Main Microplastic Types	Main Distribution Sites of Microplastics	References
Demersal fish	Ingestion of benthic organisms	3–8	Fragments	Digestive tract, liver	[51]
Pelagic fish	Misuse of microplastics in surface waters	1–3	Fibers	Digestive tract, muscle	[52]
Small yellow croaker	Accidental ingestion or food chain transfer	-	-	Liver (detection rate 5–10%)	[53,54]
Cultured oyster	Filter feeding	5–15	Fiber class (>70%)	Digestive tract	[55]
Wild oyster	Filter feeding	2–5	Fiber class (>70%)	Digestive tract	[56]
Plankton	Misuse of microplastics	1–3	Pellet class, Fiber class	Digestive tract	[57]

**Table 3:** Morphology and Migration of Microplastics.

Microplastic Type	Main Source	Morphological Characteristics	Host Organisms	Migration Type	Driving Force	Migration-Transformation Mechanism	Research Findings	References
Fiber Type	Textile fibers, fishing nets	Length of 0.5–5 mm, highly flexible, easily entangled in the digestive tract	Pelagic fish (Spanish mackerel, mackerel), bivalves (oysters, mussels)	Horizontal migration	Tidal action	Nearshore microplastics were resuspended and deposited repeatedly, forming pollution retention zones	Nearshore pollution accumulation was enhanced	[2,6,59]
Fragment Type	Degraded plastic packaging, industrial plastic waste	Sharp edges, easily caused tissue damage	Demersal fish (eel, flounder), filter-feeding organisms (bivalves, zooplankton)	Horizontal migration	Circulation transport	Microplastics were transported from northern Bohai Bay to central Bohai by the Liaodong coastal current, while the Lubei coastal current carried them southward to the Yellow Sea	Microplastics were transported across regions	[37,60]
Pellet Type	Industrial plastic raw materials, personal care microbeads	Diameter < 1 mm, easily ingested by plankton	Zooplankton (copepods, cladocerans), filter-feeding bivalves (mussels, scallops)	Vertical sinking	Biological pump	Microplastics were ingested by plankton and settled into deep water via fecal pellets	80% of microplastics were transferred through the biological pump	[61,62,63]
Pellet Type	Industrial plastic raw materials, personal care microbeads	Diameter < 1 mm, easily ingested by plankton	Zooplankton (copepods, cladocerans), filter-feeding bivalves (mussels, scallops)	Vertical sinking	Flocculation effect	Microplastics aggregated with organic matter, accelerating their sinking to the seabed	The sinking rate increased by two to three times	[64,65,66]

**Table 4:** Biological Accumulation of Microplastics in Bohai Sea Aquatic Organisms.

Category	Example Species	Ingestion Pathway	Microplastic Content	Main Particle Size	Tissue Distribution Characteristics	Biomagnification Effect	References
Filter-feeding organisms	*Crassostrea gigas*, *Ruditapes philippinarum*	Water filtration	5–15 particles/individual	-	Predominantly in the digestive tract (>90%)	-	[16,67]
Predatory organisms	*Larimichthys polyactis*, *Paralichthys olivaceus*	Ingestion of contaminated prey	2–8 particles/individual	<1 mm	15–30% were detected in the liver and gills	-	[68,69,70,71]
Benthic-feeding organisms	*Apostichopus japonicus*	Sediment ingestion	3–5 particles/g gut content	80% were fibers	Accumulated in the digestive tract	-	[72]
Zooplankton	Copepods	Accidental ingestion	0.5–2 particles/individual	50–200 μm	Found in the digestive tract	2.6–3.8	[14,72]
Primary consumers	Atheriniformes	Feeding on zooplankton	3–5 particles/individual	-	Found in the digestive tract	2.6–3.8	[22,43,73]
Top predators	*Scomberomorus niphonius*	Feeding on primary consumers	8–15 particles/individual	-	Found in the digestive tract	2.6–3.8	[74]
Cross-organ migration	*Danio rerio*	Nanoplastic penetration into the circulatory system	-	100 nm	Oxidative stress response in liver cells	-	[75]
Mammals	Seals	Ingestion of contaminated prey	-	-	Over 90% accumulated in the intestine	-	[76]

**Table 5:** Composite Toxicity of Microplastics in the Bohai Sea.

Toxicity Category	Mechanism	Example Species	Effects Observed	References
Physical Damage	Mechanical obstruction	Fish (*Lateolabrax japonicus*)	Reduced feeding efficiency by 20–40% due to digestive tract blockage	[73]
	Mechanical obstruction	Juvenile *Lateolabrax japonicus*	Survival rate decreased by 35% compared to the control group	[49,75]
	Tissue abrasion	Fish gill epithelial cells	Sharp-edged microplastics caused epithelial scratches, disrupting osmoregulation	[34,76]
	Tissue abrasion exposure	*Mytilus edulis* (Mussels)	Hemolymph pH decreased by 0.3–0.5 units due to microplastic	[77,78]
Chemical Synergistic Toxicity	Pollutant carrier	-	Microplastics had a surface area of 1.2–3.5 m^2^/g, adsorbing PAHs at 0.8–2.3 μg/g	[79]
	Pollutant carrier	-	Lead and cadmium concentrations on Bohai Sea microplastics were 45–120 mg/kg and 8–25 mg/kg, 2–4 times higher than background sediments	[17,32]
	Endocrine disruption	*Paralichthys olivaceus* (Flounder)	BPA-adsorbed polyethylene microplastics increased plasma estradiol levels by 180%, reducing gonadal development index by 22%	[6,79]

## Data Availability

The raw data supporting the conclusions of this article will be made available by the authors on request.
